# A Magnetic Nanoparticle-Doped Photopolymer for Holographic Recording

**DOI:** 10.3390/polym14091858

**Published:** 2022-04-30

**Authors:** Muhammad Irfan, Suzanne Martin, Muhannad Ahmed Obeidi, Scott Miller, Frank Kuster, Dermot Brabazon, Izabela Naydenova

**Affiliations:** 1 Centre for Industrial and Engineering Optics, School of Physics and Clinical and Optometric Sciences, College of Science and Health, Technological University Dublin, City Campus, Central Quad, Grangegorman Lower, D07 ADY7 Dublin, Ireland; d15128418@mytudublin.ie (M.I.); suzanne.martin@tudublin.ie (S.M.); 2 Advanced Manufacturing Research Centre & Advanced Processing Technology Research Centre, I-Form, School of Mechanical and Manufacturing Engineering, Dublin City University, Glasnevin, 9 Dublin, Ireland; muhannad.ahmedobeidi@dcu.ie (M.A.O.); dermot.brabazon@dcu.ie (D.B.); 3 Ambrell, B.V., 7556 BS Hengelo, The Netherlands; smiller@ambrell.com (S.M.); fkuster@ambrell.com (F.K.)

**Keywords:** photopolymer magnetic nanocomposite, volume transmission holographic gratings, magnetic nanoparticle-doped holographic gratings, holographic sensor/actuator, functionalised holograms, Denisyuk reflection hologram

## Abstract

Functionalised holograms are important for applications utilising smart diffractive optical elements for light redirection, shaping and in the development of sensors/indicators. This paper reports on holographic recording in novel magnetic nanocomposites and the observed temperature change in dry layers and liquid samples exposed to alternating magnetic field (AMF). The nanocomposite consists of N-isopropylacrylamide (NIPA)-based polymer doped with magnetic nanoparticles (MNPs), and local heating is achieved through magnetic induction. Here, volume transmission holographic gratings (VTHGs) are recorded with up to 24% diffraction efficiency (DE) in the dry layers of magnetic nanocomposites. The dry layers and liquid samples are then exposed to AMF. Efficient heating was observed in the liquid samples doped with Fe_3_O_4_ MNPs of 20 nm average size where the temperature increased from 27 °C to 64 °C after 300 s exposure to 111 mT AMF. The temperature increase in the dry layers doped with the same nanoparticles after exposure to 4.4 mT AMF was observed to be 6 °C. No temperature change was observed in the undoped layers. Additionally, we have successfully recorded Denisyuk holograms in the magnetic nanocomposite materials. The results reveal that the magnetic nanocomposite layers are suitable for recording holograms and need further optimisation in developing holographic indicators for mapping AMFs.

## 1. Introduction

New functionalised photosensitive material development is an active area of research for scientists working in the field of optical holography. The optical patterns recorded in suitably selected photosensitive materials find many applications in holography, such as holographic data storage [1,2], holographic displays [3,4,5,6,7], holographic lenses [8,9,10], holographic security [11], solar applications [12,13,14,15], interferometry [16,17], holographic polarisation optical elements [18,19,20] and holographic sensors [21,22,23,24,25,26,27]. The applicability and results for accomplishing the majority of such applications are largely dependent on the properties of the photosensitive holographic recording materials.

Among these materials, polymers are under intensive research [2,27,28]. They are preferable due to their properties, such as a self-processing nature, high sensitivity, large dynamic range, real-time image formation, high diffraction efficiency and relatively low cost in production. Another promising feature of polymers is that they can be readily functionalised for recording smart interactive holograms [29]; such holograms can then function as smart interactive optical devices for the fabrication of sensors, for example, in the detection of glucose [30,31], pH [24,32,33], pressure [29,34,35], humidity [36,37,38], temperature [29,38,39] and metal ions in water [29,40,41,42]. In the reported research literature, three approaches are investigated for achieving interactive functionalisation [27,29], each approach having its advantages and challenges. One approach among them is employing the compositional variation in the material before holographic recording. This is usually accomplished either by introducing the recording material with suitably selected monomer molecules [43,44], using silver halide chemistry to form nanoparticles during recording [25] or doping it with nanoparticles [25,27,45,46].

**Figure 1:**
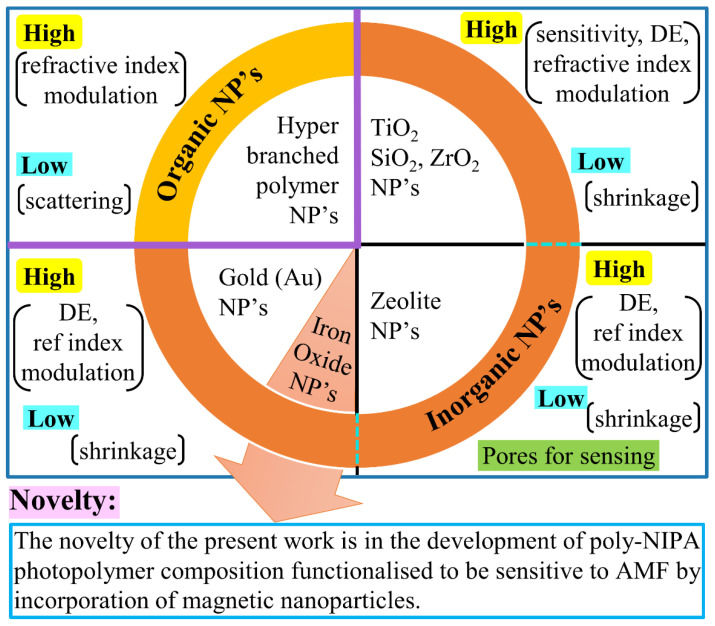
Summary schematic of the research work reported to date on the doping of nanoparticles into the holographic recording materials.

Nanoparticle-doped polymers are of significant interest because of the potential to fine-tune the material properties by independently varying the properties of the host matrix and the nanoparticles’ dopant. Thus, doping nanoparticles into the host material can offer the advantage of controlling the performance and properties of the optical device [46]. Doping with nanoparticles can also confer other properties that are beneficial to the material’s performance as a holographic recording material, such as a large dynamic range of the material, high diffraction efficiency in thinner layers, reduction in photopolymerisation shrinkage and improved grating stability [47,48]. On the other hand, there are also some challenges that can arise in the recording material due to doping it with nanoparticles. One such issue is the increased optical loss caused by scattering in the case of large differences between the refractive index of the host material and nanoparticles [47]. The idea of doping nanoparticles into the host polymer was investigated by different researchers for a wide range of nanoparticles [48]. Successful doping was carried out for organic nanoparticles, such as hyperbranched polymer NPs [49,50], and different inorganic nanoparticles, e.g., TiO_2_ [51,52], ZrO_2_ [53,54], SiO_2_ [55,56,57], gold (Au) [58], Zeolite NPs [46,47,48,59,60], in each case either improving the properties of the holographic recording material or aiming to achieve an application, as shown in Figure 1. Zeolite NPs are particularly important among the doped NPs as, due to their porous structure, they can selectively adsorb/absorb certain molecules and thus can be used to introduce sensitivity in the photopolymer system for specific analytes [47,60,61]. Work was carried out in this regard in developing a holographic sensor for toluene by doping zeolite Beta NPs in photopolymer [62]. In another research study, an irreversible holographic indicator was developed for humidity by doping photopolymer with AIPO-18 zeolite NPs [63].

Other research studies reveal that the incorporation of certain nanoparticles, such as gold [64] and magnetic nanoparticles [65,66], can trigger a process in the materials (e.g., heating) when they are exposed to external stimuli. Magnetic nanoparticles are well-known for remote-controlled induction heating and are extensively studied for a wider range of applications, such as magnetic hyperthermia [66], drug delivery [66] and actuators [67,68]. In all these cases, local heating can be achieved in the material through magnetic induction phenomena under an alternating magnetic field (AMF) due to the presence of MNPs.

Following the successful research carried out previously on polymer nanocomposites containing a variety of nanoparticles, the work presented here uses a similar approach to the functionalisation of the photopolymer for holographic recording but this time incorporates magnetic nanoparticles (MNPs). The material composition used in this study is unique, which consists of N-isopropylacrylamide (NIPA)-based photopolymer doped with MNPs, such as Fe_2_O_3_ Alpha with a particle size range of 20–30 nm and Fe_3_O_4_ with an average particle size of 20 nm. The host material NIPA-based photopolymer in its pure form is studied for holographic recording and has sensitivity to elevated temperature [44,69,70,71]. The photosensitive mixture contains a monomer (N-isopropyl acrylamide (NIPA)), a cross-linker (N,N-Methylene bisacrylamide (BA)), an initiator (N-Phenylglycine (NPG)), a sensitising dye (Erythrosine B (Er B)), a free radical scavenger (Glycerol) and a chain transfer agent (citric acid (CA)). This photopolymer composition was selected because, after holographic recording, a spatially distributed PolyNIPA polymer is created, which leads to a spatial variation in the refractive index of the layer. This variation causes the diffraction of a probe beam when incident on the recorded grating. PolyNIPA is known for its phase transition from a hydrophilic to a hydrophobic state at a lower critical solution temperature (LCST), which, for pure PolyNIPA, is 32 °C. The LCST can be controlled by the amount of cross-linker present in the mixture. The sensitivity of PolyNIPA to temperature variations in a range including the LCST make it a very good choice for development of a polymer material that is suitable for recording holograms and, at the same time, can be functionalised to produce holograms sensitive to an external alternating magnetic field (AMF). The long-term objective of this research is to develop a larger-area polymeric layer containing an array of holograms that can change their colour when exposed to an AMF. The different colour change can then be used to map the magnetic field strength. This will be particularly useful for the characterisation of the MRI medical equipment magnetic field or for the characterisation of more complex magnetic fields. The concept for the development of a holographic material sensitive to magnetic fields is as follows: it is expected that the presence of MNPs will lead to the elevation of the polymer temperature when an AMF is applied. Energy from the magnetic field will dissipate to heat energy, then transfer from MNPs to the host polymer material and increase its temperature locally [72]. The local change in temperature caused by MNPs will then lead to shrinkage of the polymer layer and is expected to cause changes in the characteristics of the recorded hologram in the polymer nanocomposite layer. Here, it is worth mentioning that we have previously reported an initial study on holographic recording in photopolymer doped with different MNPs, Fe_2_O_3_-Gamma 30 nm, concentrations of 0.5 and 1% wt./wt., where transmission gratings with a maximum diffraction efficiency of about 18% were recorded in a layer with an MNPs concentration of 0.5% wt./wt. [73]. In this work, the study of holographic recording in magnetic nanocomposites is further extended, and it is found that holograms can be recorded in layers with even higher concentrations (2, 5 and 10% wt./wt.) of another type of MNPs, Fe_2_O_3_ Alpha 20–30 nm. We have also investigated the holographic recording in magnetic nanocomposites containing MNPs Fe_3_O_4_ with an average particle size of 20 nm. It is anticipated that obtaining a holographic recording in a higher concentration of MNPs is required as the higher concentration of MNPs in the nanocomposite helps in achieving higher temperature change upon exposure to an AMF. Additionally, we have demonstrated that Denisyuk reflection holograms can be recorded in magnetic nanocomposite material having MNPs Fe_2_O_3_-Alpha with a particle size range of 20 to 30 nm.

To the best of our knowledge, the doping of NIPA-based photopolymer with MNPs for holographic recording in photopolymer magnetic nanocomposite layers and, particularly, the study of the possibility of local heating achieved in their liquid (solution) and solid samples (dry layers) through magnetic induction under an AMF are reported here for the first time.

## 2. Theoretical Background

Holographic recording is typically carried out by recording the interference pattern of two collimated coherent beams in a photosensitive recording material. During recording, the optical properties of the material vary according to a periodic interference pattern of light. The recorded holographic gratings can be categorised as thin (surface/plane) and thick (volume) gratings, their properties differ and their uses vary according to the application sought. The thick (volume) gratings are of particular interest; they are recorded throughout the depth of a photosensitive holographic recording material and obey Bragg’s condition by producing a single diffraction order beam when reconstructed with a probe beam incident at Bragg angle. The work presented here is based on thick (volume) gratings, where the period (and, thus, the special frequency) of gratings is typically achieved by adjusting the angle between the recording beams according to Equation (1).

(1) $$\lambda =2n_{o}\Lambda \sin\theta$$

Here, λ is recording beam wavelength, ո_o_ is the effective refractive index of the recording medium (photopolymer), Λ is grating period, θ is half of the angle between the two recording beams inside the medium.

The visibility, V, of the interference fringes, in terms of reference beam intensity, I_r_, and object beam intensity, I_o_, can be expressed by Equation (2),

(2) $$V=\frac{2\sqrt{I_{r}}I_{o}}{I_{r}+{\;I}_{o}}$$

where visibility is maximum when the two recording beams have equal intensities.

The Kogelnik’s coupled wave theory (KCWT) [74] provides an analytical expression for the diffraction efficiency (DE) of thick (volume) gratings. According to KCWT, the maximum DE of unslanted transmission VPHGs at Bragg’s incidence for s-polarised light is determined by Equation (3). The refractive index modulation achieved can be calculated by rearranging Equation (3), as given in Equation (4).

(3) $$\eta =\sin^{2}\left(\frac{\pi d\Delta n}{\lambda_{p}{\cos\theta }_{B}}\right)$$

(4) $$\Delta n=\frac{\cos\left(\theta_{B}\right)\lambda_{p}\sin^{-1}\sqrt{\eta }\;}{\pi d}$$

Here, η is the DE of the recorded transmission VPHGs, ∆n is refractive index modulation (RIM) achieved during recording, d is thickness of the grating, λ_p_ is probe beam wavelength and θ_B_ is the Bragg angle of incidence inside the medium.

The sensitivity of the diffraction efficiency to detuning from Bragg angle is characterised by measuring the Bragg angular selectivity curve. By measuring and fitting the Bragg selectivity curve, it is possible to estimate the thickness of the recorded volume grating structure. The Bragg selectivity curve is given by Equation (5).

(5) $$\eta =\sin^{2}{\left(\frac{\Phi^{2}+\chi^{2}}{1+\chi^{2}/\Phi^{2}}\right)}^{1/2}$$

where $\Phi =\frac{\pi d\Delta n}{\lambda_{p}{\cos\theta }_{B}}$ is the phase difference between the transmitted (zero order) and the diffracted beam and $\chi =K\frac{\Delta \theta_{B}d}{2}$ is the phase detuning caused by deviation from Bragg angle Δθ_Β_ and K is the grating vector magnitude. 

## 3. Materials and Methods

### 3.1. Materials

Polyvinyl alcohol (PVA), N-isopropyl acrylamide (NIPA), N,N-Methylene bisacrylamide (BA), N-Phenylglycine (NPG), Erythrosine B (Er B) and citric acid (CA) were purchased from Sigma-Aldrich, St. Louis, MO, USA. Glycerol was purchased from Fisher Scientific, Hampton, NH, USA, while Fe_3_O_4_ 20 nm and Fe_2_O_3_ 20–30 nm MNPs were purchased from GetNanoMaterials, Saint-Cannat, France. All chemicals were used without further modification.

The stock solution of Polyvinyl alcohol (PVA) and Erythrosine B (Er B) sensitising dye was prepared as previously reported [71]. For PVA solution (10% wt./vol), 10 g of PVA powder was dissolved in 100 mL of deionised (DI) water through magnetic stirring and heating at 75 °C. A dye solution of concentration (0.11% wt./vol) was obtained by dissolving 0.11 g of Er B dye in 100 mL of DI water through magnetic stirring at room temperature.

### 3.2. Preparation of Liquid (Solution) of Nanocomposite (PVA Doped with MNPs)

Liquid (solution) samples of PVA-doped nanocomposites are prepared by doping PVA (10% wt./vol) solution with MNPs. Two types of MNPs, Fe_3_O_4_ 20 nm and Fe_2_O_3_ 20–30 nm, having the different chemical composition but similar sizes, are separately doped into PVA solution. This experiment was carried out to investigate the role of MNPs in their ability to show hyperthermal effect under an AMF. Here, MNPs are distributed in PVA solution by using Ultrasonic probe Sonicator (Sonics, Model: GEX 750). In this case, PVA is used as a host material instead of standard NIPA-based photopolymer because the latter is sensitive to light due to presence of sensitising dye (Er B) and can polymerise as well as being sensitive to temperature. Since the samples preparation lab (Centre for Industrial and Engineering Optics, Technological University Dublin, Dublin, Ireland) and AMF testing facility (Ambrell, B.V., Hengelo, The Netherlands) are based at different locations, to avoid the possibility of light-initiated polymerisation, exposure to uncontrolled environmental conditions during samples transit and their impacts, PVA solution is only used here. The viscosity of the PVA and that of the photopolymer solution are very similar at about 52 millipascal second (mPa·S). The finally prepared nanocomposite solutions (PVA doped with Fe_3_O_4_ with average size 20 nm and PVA doped with Fe_2_O_3_ with average size 20–30 nm) are labelled as composition (A) and (B), respectively, as in Table 1.

### 3.3. Preparation of Pure NIPA-Based Photopolymer

The preparation procedure of undoped NIPA-based polymer for holographic recording has been previously reported [44,71]; here, the same approach was adopted to prepare pure poly-NIPA polymer composition C by thoroughly mixing all the components in a glass beaker with magnetic stirring for 1–2 h at laboratory conditions (relative humidity, RH 32 ± 4% and temperature 20 ± 2 °C) until a homogeneous solution was obtained, labelled as composition (C) in Table 2.

### 3.4. Polymerisable Magnetic Nanocomposites

#### 3.4.1. Selection of Suitable Magnetic Nanoparticles (MNPs) and Host Polymer

In this work, the introduction of magnetic nanoparticles in NIPA-based photopolymer has been investigated for the first time for holographic recording in reflection mode and nanoparticle-doped polymer samples were studied under an AMF to see the possibility of local heating through magnetic induction phenomena. N-isopropylacrylamide (NIPA)-based photopolymer was chosen as host material due to its temperature-sensitive nature [44,69,70,71], where the presence of MNPs will help in the elevation of the polymer temperature when an external AMF is applied. The search for suitable MNPs aimed at identifying nanoparticles that have magnetic properties and are, at the same time, compatible with the photopolymer used for recording holograms. Throughout the search, it was noticed that iron oxide magnetic nanoparticles have numerous advantages over other magnetic materials, e.g., Nickel, Cobalt. Iron oxide MNPs are relatively easy to synthesise, they are biocompatible, non-toxic, chemically rather stable and can be superparamagnetic [75]. Additionally, the selection was guided by reports from the literature [67,68,76,77], describing the use of MNPs in biomedical and actuator type applications. As described in previous research [76,77,78], various characteristics of MNPs, such as chemical composition, size and structure, play a substantial role in their ability to show hyperthermal effect in magnetic field. Therefore, at the same time, MNPs should be large enough in size to cause heat generation when the photopolymer nanocomposite material is exposed to AMF and small enough to achieve a reasonable optical quality of nanocomposite layer used for holographic recording. Among all iron oxide MNPs, magnetite (Fe_3_O_4_) and maghemite (Fe_2_O_3_) were identified as the two main forms that are most commonly used MNPs, whereas magnetic materials such as Nickel and Cobalt are susceptible to oxidation and are toxic; hence, they were of little interest [79,80,81].

#### 3.4.2. Selection of Suitable Dispersion Approach and Media Used for MNPs Dispersion to Achieve Good Quality Dry Layers

Ultrasonic probe Sonicator (Model: GEX 750) was used in order to ensure that nanoparticles distribute homogeneously throughout the polymer solution. It was found that probe sonication worked very effectively in distributing MNPs and 30 s duration of sonication was found sufficient. As probe sonication can also cause heating of the solution/liquid that is sonicated, it was observed that sonication of the MNP-doped NIPA-based polymer solution for 30 s leads to a temperature increase of up to 30 ± 0.5 °C. This temperature increase could cause a change or local polymerisation of the NIPA-based photopolymer solution as the pure poly (NIPA) polymer has lower critical solution temperature (LCST) at 32 °C [82] and the achieved temperature after sonication was close to this value. For that reason, the nanoparticles were first dispersed in PVA and only then added to the photopolymer liquid.

Firstly, de-ionised water was used for dispersing the MNPs in a liquid, but it was noticed that MNPs suspension was not stable as they quickly settled down to the base of container as soon as the sonication stopped. In the next step, the nanoparticles were dispersed in PVA solution of concentration 10% wt/vol. The layers obtained from the final photosensitive nanocomposite are shown in Figure S1. It is clearly seen that the layers prepared with the MNPs dispersed in the 10% wt./vol. PVA were of good optical quality and, thus, selected for study in holographic recording.

#### 3.4.3. Preparation of Polymerisable Magnetic Nanocomposites

**Figure 2:**
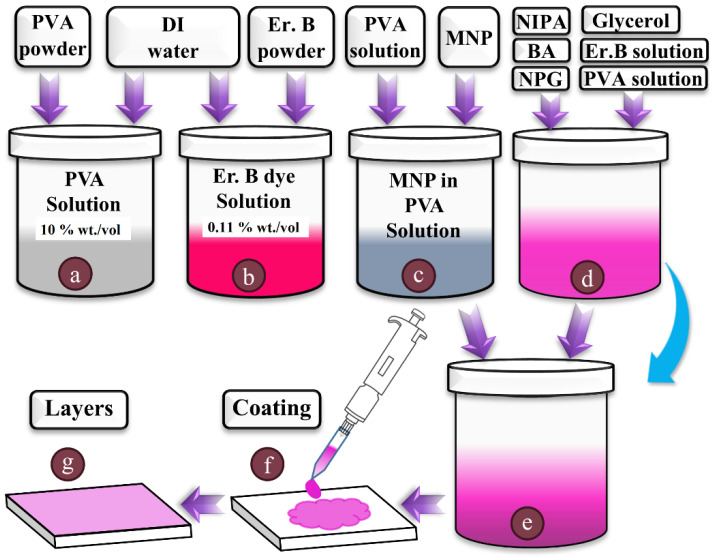
Preparation of the liquid samples and nanocomposite layers. (**a**) PVA solution, (**b**) Er. B dye, (**c**) MNP are dispersed in PVA solution, (**d**) pure NIPA-based polymer, (**e**) photopolymer nanocomposite achieved by mixing materials from step-(**c**) and step-(**d**), (**f**) coating of nanocomposite solution, (**g**) finally prepared nanocomposite layer.

In order to avoid temperature rise of the photosensitised nanocomposite solution during sonication, the nanoparticles were first dispersed in PVA 10% wt./vol. and then added to the photopolymer solution. As previously reported [44], in the preparation of optimised NIPA-based photopolymer, 16 mL of PVA 10% wt./vol. was used along with other constituents. Half (8 mL) of the PVA 10% wt./vol. was mixed thoroughly with NIPA, BA, NPG, Er B, Glycerol, CA according to the compositions mentioned in Table 2, while the MNPs were dispersed in the remaining 8 mL of PVA through probe sonication. The 30 s probe sonication was carried out in three steps each of 10 s active sonication, followed by a cooling (no-sonication) period of 15 s. As shown in Figure 2, the two solutions (2c)-suspension of MNPs in PVA and (2d)-NIPA-based photopolymer were than mixed to achieve polymerisable magnetic nanocomposite solutions of different compositions suitable for recording in transmission (D-Trans) or reflection (E-Reflect) mode, as described in Table 2.

### 3.5. Preparation of Samples

Liquid samples of nanocomposite (PVA doped with MNPs) were prepared for testing the heating efficiency of MNPs in the AMF. Macro cuvettes (dimensions 4.5 cm × 1.25 cm × 1.25 cm) are separately filled with up to 4 mL solution of each composition (A), (B) and PVA only and the cuvettes were covered with a lid.

Dry layer samples were prepared from compositions (C), (D) and (E) for holographic recording. Layers were made from pure NIPA photopolymer composition (C), nanocomposite composition (D) and composition (E) by depositing and evenly coating the photopolymer solution on microscopic glass slide (dimension 7.6 cm × 2.6 cm × 0.1 cm), followed by drying for 8–10 h in dark room at relative humidity, RH 32 ± 4% and temperature 20 ± 2 °C. For composition (C), (D), layers of thickness 90 ± 8 µm were obtained by depositing 1.5 mL solution on microscopic glass slide. In case of composition (E), layers of thickness 44 ± 5 µm were, respectively, obtained by separately depositing 0.4 mL solution on the microscopic glass slide with the help of a micropipette and by drying the samples on a levelled marble surface. In samples of composition (D), (E) containing MNPs, the doped MNPs are physically entrapped inside the host material and do not interact chemically with the photopolymer. It is found in previous research that MNPs can be physically entrapped in the polymer network and, by free radical polymerisation, a magnetic hydrogel nanocomposite can be obtained [83]. The physical pictures of the magnetic nanocomposite layers used for holographic recording in transmission mode are shown in Figure S1 (Supplementary Materials) and layers used in reflection mode of recording are shown in Figure S2.

### 3.6. Holographic Recording of Transmission Gratings

**Figure 3:**
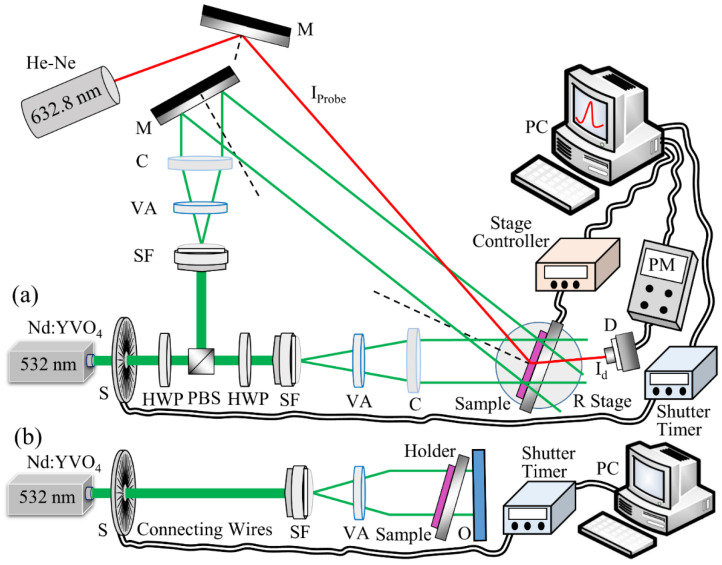
Experimental setups for recording (**a**) volume transmission holographic gratings and (**b**) Denisyuk reflection holographic gratings. Where S: electronic shutter; HWP: half wave plate; PBS: polarising beam splitter; SF: spatial filter; VA: variable aperture; C: collimator; M: mirror; PP: photopolymer; D: photodetector; PM: power metre; PC: computer; O: object.

Volume unslanted transmission holographic gratings (VTHGs) were recorded in the dry layer samples of pure NIPA-based photopolymer (composition C) and magnetic nanocomposites (composition D) by using the setup shown in Figure 3a. The two collimated beams were obtained by splitting laser light from Nd:YVO_4_ laser (532 nm) with a polarising beam splitter (PBS) and a total angle between the recording beams of 24.57° to create an interference pattern of spatial frequency of approximately 800 lines/mm (recorded grating period was 1.25 µm). Maximum visibility of the interference fringes was ensured by establishing same polarisation state (s-polarised) for both recording beams; for this purpose, an additional half wave plate (HWP) was used in the path of one of the recording beams. During the holographic recording, dry layer samples (composition C, D) were exposed to the interference pattern of two beams having diameter of 13 ± 1 mm.

Real-time development of the holographic gratings was monitored by measuring its diffraction efficiency (DE) growth curves. Since the host polymer used here has negligible absorption at 632.8 nm, to ensure that the recording process was carried out appropriately, a helium-neon (He-Ne) laser of 632.8 nm wavelength was used as probe beam to study the real-time recording of holograms. After recording, Bragg selectivity curves of the gratings were obtained by rotating the samples placed on a computer-controlled rotational stage (Newport ESP300), thus varying the probe beam incident angle by ±3° from Bragg angle. This was carried out in order to observe the dependence of diffracted light intensity on the incident angle of probe beam. For acquiring real-time DE growth as well as Bragg curves, the first order diffracted beam intensity (I_d_) was monitored with an optical power metre (Newport Model 840), and computer software (LabVIEW) was used to plot the acquired data of DE in real time. The DE value was calculated as the ratio of the diffracted beam intensity (I_d_) and the incident beam intensity (I_i_). The mechanical stability of the optical setups was ensured by using a floated optical table (Newport RS 4000).

### 3.7. Holographic Recording of Denisyuk Reflection Gratings

Denisyuk reflection holographic gratings were recorded in pure NIPA and magnetic nanocomposites material (composition E) with laser light from Nd:YVO_4_ laser (532 nm) as in setup in Figure 3b. For recording, the photopolymer layer was kept very close to the object and tilted slightly with a small angle (approx. 5°) to avoid the overlap of reflected and diffracted beam in readout. During recording, layers were exposed for 100 s to the interference pattern formed by the incident and reflected beams with total recording intensity 35 mW/cm^2^.

### 3.8. Testing Samples in Alternating Magnetic Field (AMF)

To study the response of undoped and doped samples and their heating capability in the AMF, different experimental setups/equipment were used as described below.

#### 3.8.1. AMF Setup Used for Testing Liquid (Solution) Samples

The following sections present the results obtained from heating capability of magnetic nanocomposite liquid samples through magnetic induction. Liquid samples of composition A and B were prepared and investigated to test whether the magnetic induction heating is possible in the samples used and the maximum temperature that can be achieved. Additionally, magnetic induction setups of different powers and MNPs Fe_3_O_4_ with average size 20 nm, Fe_2_O_3_ Alpha with average size 20–30 nm having similar sizes but different chemical compositions are used to investigate the impact of equipment power and magnetic field strength and MNPs on the heating capability.

##### Low-Power Setup for Testing Liquid (Solution) Samples

**Figure 4:**
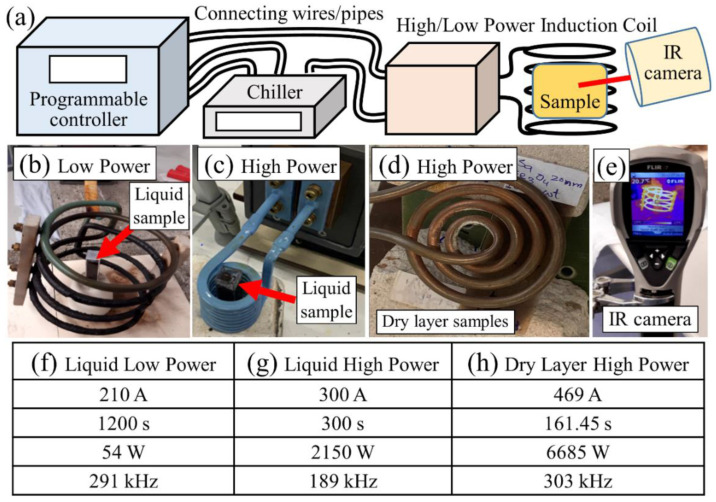
Setups used: (**a**) schematic representation of the experimental setup, (**b**) setup of low-power induction heating system for AMF test of liquid samples (tested at DCU university lab), (**c**) setup of high-power induction heating system for AMF test of liquid samples (tested in Ambrell lab), (**d**) setup of high-power induction heating system for AMF test of dry samples (tested in Ambrell lab), (**e**) IR camera used to monitor changes in the samples’ temperature upon exposure to AMF. The exposure conditions for (**b**–**d**) are labelled, respectively, as (**f**–**h**) in table.

The heating capability of magnetic nanocomposite liquid (solution) samples composition A and B was investigated in low-power experimental setup (Easy Heat 2.4 kW Induction Heating System, Ambrell) shown in Figure 4. In Figure 4a, the schematic representation of the experimental setup is shown, while Figure 4b is a zoom-in picture of sample inside the magnetic field coil in low-power setup. Liquid samples containing 4 mL solution of each composition A, B separately in the macro cuvette container and an additional sample of pure PVA 10% w/v only were exposed to AMF with conditions achieved (current 210.0 Amps, time duration 1200 s, power 54 W and frequency 291 kHz) shown in column (g) in the table (Figure 4). Here, the current and exposure time duration are the two inputs that can be inserted in the controller; the current was kept at its maximum value for the system used, while power is achieved as output. The maximum magnetic field strength achieved at the sample location with this system was 0.06 mT. An infrared (IR) camera fixed at specific distance was used to monitor temperature of the samples throughout exposure to AMF; the camera position was not moved/modified throughout the experiment. During the first 5 min, an IR camera image was taken every minute and then after every five minutes.

##### High-Power Setup for Testing Liquid (Solution) Samples

Doped liquid sample composition was exposed to high-power magnetic induction in the EASYHEAT 0224 induction heating system (Ambrell) as shown in Figure 4c. The sample contained 4 mL solution of composition A in the macro cuvette container and was exposed to AMF parameters (current 300.0 Amps, time duration 300 s, power 2150 W and frequency 189 kHz) shown in column (g) in the table (Figure 4). The maximum magnetic field strength achieved with this system was 111 mT. Temperature of the samples was monitored by an IR camera.

#### 3.8.2. AMF Setup Used for Testing Dry Layer Solid Samples

The heating capability of dry layer samples through magnetic induction phenomena was investigated by exposing both the undoped (composition C) and doped (composition D) samples to AMF. The sample used here was made of NIPA-based photopolymer doped with Fe_3_O_4_ with average size 20 nm MNPs having 10% wt/wt concentration. The equipment used was an EASYHEAT 8310 induction heating system (Ambrell) shown in Figure 4d. Both undoped and doped dry layer samples were placed over two solid concrete blocks such that one end of samples is on first block while the other end on second block. The pancake-type induction coil was adjusted on top of the samples at a distance of 5 mm from them, and they were exposed to AMF parameters (current 469.0 Amps, time duration 161.45 s, power 6685 W and frequency 303 kHz) shown in column (h) in Figure 4d. The magnetic field strength at the plane of the samples in a point above the centre of the pancake coil was estimated to be 4.4 mT following an approach described in Ref. [84]. An IR camera was used to monitor temperature of the samples.

## 4. Results and Discussion

### 4.1. Holographic Recording in Dry Layers of Pure Photopolymer and Nanocomposites

#### 4.1.1. Transmission Gratings

##### Effect of Nanoparticle Concentration in Layers Doped with Fe_2_O_3_ Alpha with Average Size of 20–30 nm MNPs

**Figure 5:**
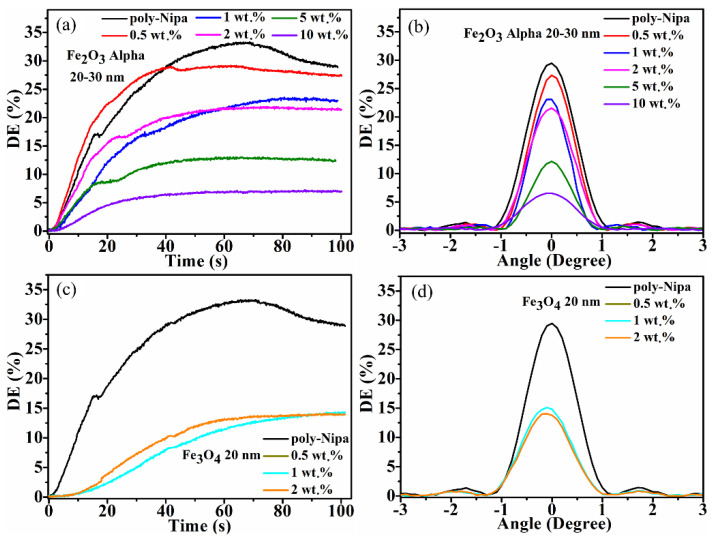
Characterisation of unslanted transmission holographic gratings. (**a**,**c**) Real-time growth curves and (**b**,**d**) Bragg selectivity curves. Gratings recorded in pure poly-NIPA photopolymer and nanocomposite doped with 0.5, 1, 2, 5, 10% wt./wt. MNPs Fe_2_O_3_ Alpha (average size 20–30 nm) (**a**,**b**) and gratings recorded in nanocomposite doped with 0.5, 1, 2% wt./wt. MNPs Fe_3_O_4_ (average size −20 nm) (**c**,**d**). The recording intensity was 6.5 mW/cm^2^ and layer thickness 90 ± 8 µm.

This section presents the results obtained by recording VTHGs using the setup shown in Figure 3a. The holographic recording capability of pure NIPA-based photopolymer and photopolymer nanocomposites (NIPA-based photopolymer doped with MNPs) was investigated by real-time monitoring of the recording process. The real-time DE growth curves obtained under the same conditions (recording intensity 6.5 mW/cm^2^) for pure NIPA-based photopolymer and photopolymer nanocomposites doped with different concentrations (0.5, 1, 2, 5, 10% wt./wt.) of Fe_2_O_3_ Alpha (average size 20–30 nm) in the dry layers are shown in Figure 5. There is a clear relationship between the concentration of the dopant and the rate of growth of the diffraction efficiency, with the poorest growth occurring in the layers with the highest concentration, which can be ascribed to increased scattering, as evident from the absorption spectra of the samples (Figure S3). Under the same recording conditions, the undoped layers reach saturation after 60 s of recording and reach up to 30% DE, while the doped layers saturate sooner and reach comparatively lower DE values. The lower diffraction efficiency achieved in the nanocomposite layers may possibly be due to increased scattering due to the presence of MNPs. Nevertheless, the nanocomposite layers having 5 and 10% wt./wt. of Fe_2_O_3_ Alpha MNPs record up to 9% and 5% DE, respectively. The surface morphology of the recorded grating structures was studied by utilising atomic force microscopy (AFM), and the obtained results are shown in Figure S4 for the gratings recorded in the magnetic nanocomposite layer having MNPs Fe_2_O_3_-Alpha. The grating period obtained is about 1.25 μm, matching the grating period value for the recording spatial frequency of approximately 800 lines/mm used in transmission holographic recording. The surface relief amplitude was in the order of a few nanometres; thus, the contribution of the surface relief grating to the diffraction efficiency can be neglected.

##### Effect of Nanoparticle Concentration in Layers Doped with Fe_3_O_4_ with Average Size of 20 nm MNPs

The results of the holographic recording in the photopolymer nanocomposites doped with MNP Fe_3_O_4_ (concentration 0.5, 1, 2% wt./wt.) in the dry layer are shown in Figure 5c,d. Recording in the samples doped with 5 and 10% wt./wt. was not attempted due to the very poor optical quality of the layers. Here, the maximum DE obtained is up to 16% and 15% in case of 0.5 and 1% wt./wt. concentration of Fe_3_O_4_ MNPs.

**Figure 6:**
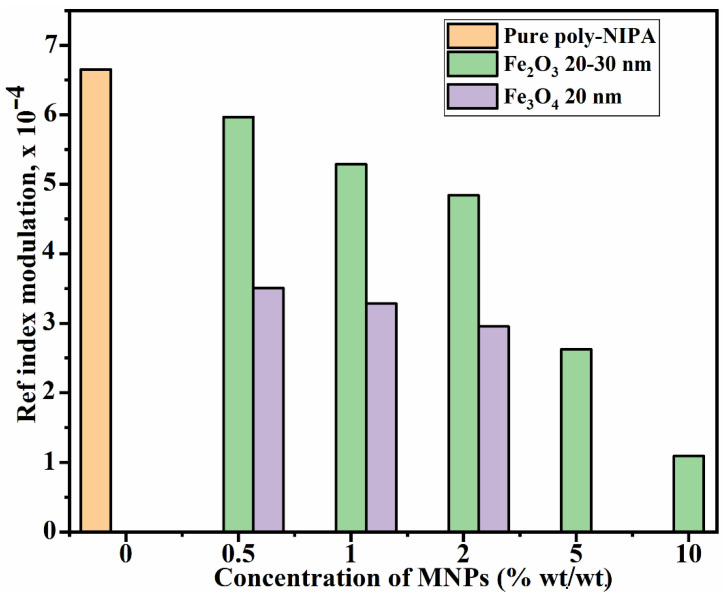
Refractive index modulation (RIM) vs concentration of MNPs in the dry layer of nanocomposites having Fe_2_O_3_ Alpha (average size 20–30 nm) and Fe_3_O_4_ (average size 20 nm).

For the transmission holographic gratings in Figure 5, the maximum refractive index modulation obtained was calculated with the help of Equation (4) and plotted versus the concentration of the MNPs in the layer. The obtained results are shown in Figure 6, and it is found that, among all the samples studied here for holographic recording, the undoped NIPA photopolymer achieves the highest refractive index modulation (6.6 × 10^−4^). For the Fe_2_O_3_ Alpha MNPs, the refractive index modulation decreases with an increase in the MNPs concentration in the layer. A similar trend is observed for the Fe_3_O_4_ MNPs.

From these results, it can be concluded that holographic recording is possible in the photopolymer nanocomposite material doped with MNPs but with some limitations on the concentration. Further comprehensive study is needed to increase the achieved diffraction efficiency in layers doped with higher concentrations of MNPs. For example, optimisation of the size of the MNPs and the recording conditions (e.g., spatial frequency, recording intensity, exposure time) and layer thickness may lead to higher diffraction efficiency gratings.

#### 4.1.2. Denisyuk Reflection Gratings

**Figure 7:**
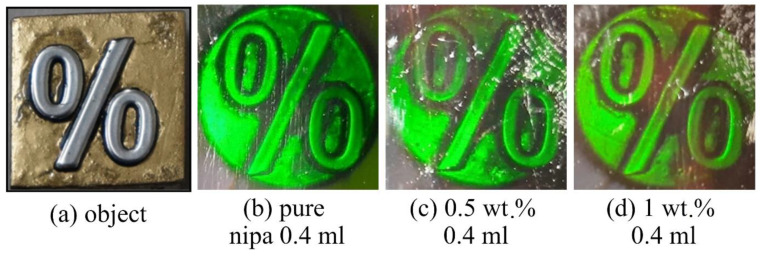
Results of Denisyuk reflection recording in NIPA-based photopolymer and magnetic nanocomposites doped with MNPs Fe_2_O_3_ Alpha (average size 20–30 nm). (**a**) A photograph of the object, (**b**) hologram recorded in pure NIPA layer (0% wt./wt. concentration of MNPs), (**c**) hologram recorded in magnetic nanocomposite layer having 0.5% wt./wt. concentration of MNPs, (**d**) hologram recorded in magnetic nanocomposite having 1% wt./wt. concentration of MNPs. The layer thickness was about 44 ± 5 µm.

The photographic images of the object (mathematical operator percentage sign (%)) and the Denisyuk reflection holograms recorded in the pure NIPA-based photopolymer and magnetic nanocomposite are shown in Figure 7. The reflection holograms typically have higher spatial frequency of the order of a few thousands of linepairs/mm. Thus, showing the capability of recording Denisyuk reflection holograms in the magnetic nanocomposite material is investigated here because it is one way to indicate that the magnetic nanocomposite material is capable of recording holographic gratings of high spatial frequency. The Denisyuk reflection holograms can be observed after illumination with a white light source, and, because they do not require laser light during reconstruction, they are relatively easier to be used as visual indicators.

Photographs of the object (Figure 7a) and holograms recorded in the undoped layer (Figure 7b) in a layer doped with 0.5% wt./wt. and 1% wt./wt. Fe_2_O_3_ Alpha MNPs are presented in Figure 7c,d, respectively. The layers were of a thickness about 44 ± 5 µm. Attempts were also made to record in thicker layers and in layers with higher concentrations of MNPs (2% wt./wt.), but they did not produce good holograms and their photographic images were not of sufficient quality; hence, their images are not included here.

By comparing the Denisyuk hologram recorded in the pure NIPA-based photopolymer layer (Figure 7b) with those recorded in magnetic nanocomposites (Figure 7c,d), it can be seen that a clearer and brighter hologram is recorded in the pure NIPA-based photopolymer layer. Nevertheless, these results show that holographic recording of reflection holograms is possible in our magnetic nanocomposite material. The issue of the lower-quality Denisyuk hologram is possibly because of the increased scattering in the nanocomposite layers, which leads to a decrease in the intensity of the object beam that passes through the nanocomposite layer twice. Any difference between the intensity of the object beam and reference beams eventually leads to a decrease in the visibility of the interference fringes (Equation (2)) recorded in the layer.

### 4.2. Exposure to Alternating Magnetic Field

#### 4.2.1. Low-Power Setup Liquid Samples

**Figure 8:**
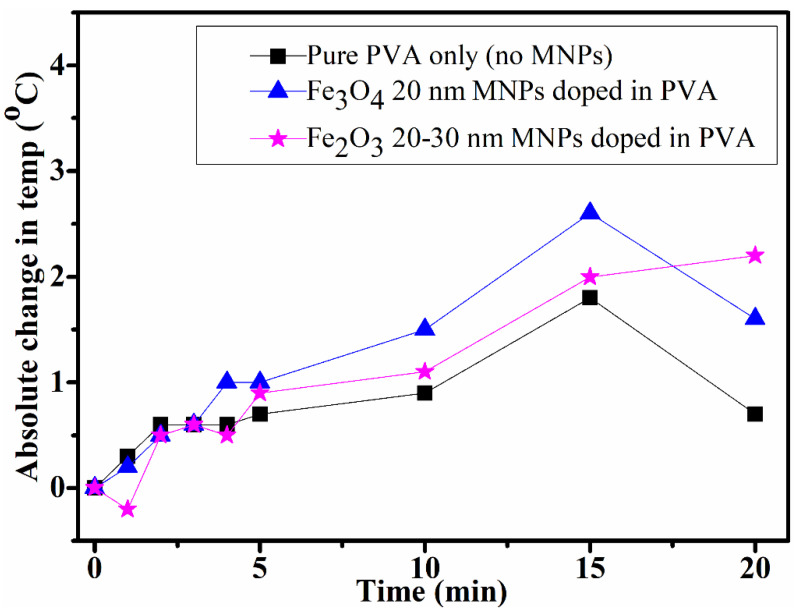
Results obtained by exposing to low-power induction system in DCU lab; absolute change in temperature (°C) vs. time (min).

The heating capability of the magnetic nanocomposite liquid samples was first investigated in a low-power experimental setup (Easy Heat 2.4 kW Induction Heating System, Ambrell) shown in Figure 4b. The temperature was recoded after each minute during the first 5 min and then after each 1 min via the thermal image camera (see Figure 8). During exposure to the AMF, a slight increase in the absolute temperature (°C) was recorded. The magnetic nanocomposites samples showed a slightly higher temperature rise compared to the PVA sample. The sample experiencing the larger temperature rise (containing Fe_3_O_4_ MNPs) was selected to be tested further in a higher-power AMF setup.

#### 4.2.2. High-Power Setup Liquid Samples

In the next experiment, doped liquid samples of composition A (containing MNPs Fe_3_O_4_) were exposed to high-power magnetic induction in the EASYHEAT 0224 induction heating system (Ambrell Lab), as shown in Figure 4c. The sample contained 4 mL solution of composition A in the macro cuvette container. The temperature of the samples was monitored by the thermal image camera.

**Figure 9:**
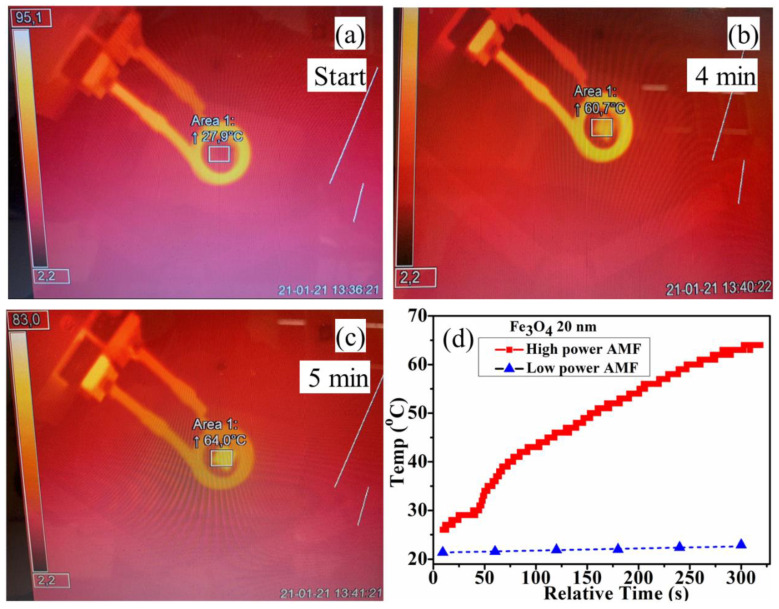
Experimental results of exposing solution samples (Fe_3_O_4_ MNPs doped in PVA) to AMF. IR camera images taken at the start of the experiment (**a**), after 4 min (**b**) and after 5 min (**c**), temperature change of Fe_3_O_4_ doped layers during exposure to AMF (**d**).

The composition (A) sample was exposed to AMF (current 300.0 Amps, time duration 300 s, power 2150 W and frequency 189 kHz) with maximum magnetic field strengths of 111 mT, and the obtained results are shown in Figure 9. During exposure to the AMF, a rise in the temperature of the sample was noticed, increasing from 27.9 °C (Figure 9a) to 64.0 °C (Figure 9c) within 5 min of exposure. The increase in the temperature versus exposure time to AMF is shown in Figure 9d. Although the sample used here and in Figure 8 (blue triangle symbols) was of the same composition (A), here, a much larger increase in temperature of about 36 °C in 300 s (5 min) after exposure to AMF was achieved.

#### 4.2.3. Dry Layer Samples

**Figure 10:**
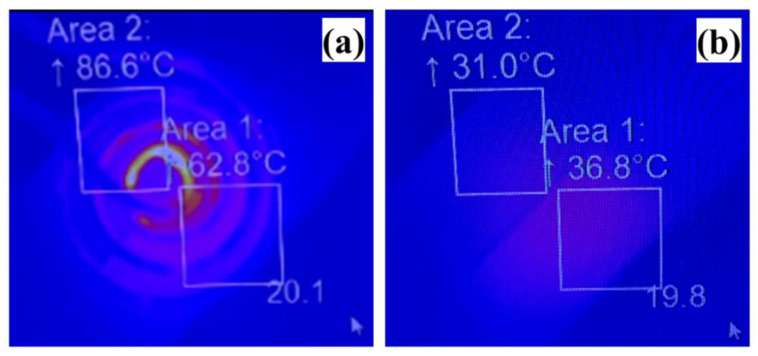
Exposing the dry layer samples (Fe_3_O_4_ 20 nm doped in poly-NIPA photopolymer) to AMF; the obtained results are shown in (**a**) during exposure and (**b**) after exposure to AMF.

The dry layer samples of the undoped NIPA-based photopolymer and nanocomposite layer containing Fe_3_O_4_ MNPs having a 10% wt./wt. concentration were exposed to AMF, as shown in the setup in Figure 4d. The conditions during the AFM exposure were: current 469.0 Amps, time duration 161.45 s, magnetic field strength 4.4 mT, power 6685 W and frequency 303 kHz, and an infrared IR camera/system was used to monitor the temperature of the samples. The results obtained during exposing samples to AMF and after exposure are shown in Figure 10a,b, respectively. In Figure 10a, area 2 is where the leads of the coil go out to the induction system and underneath the coil is the undoped sample (NIPA-photopolymer), while the doped sample (nanocomposite having MNPs Fe_3_O_4_) is towards area 1. During exposure to the AMF (induction coil above the samples), the temperature of area 1 is 62.8 °C and of area 2 is 86.6 °C; they are the highest temperatures within the respective square area, whereas 20.1 °C is the table temperature on which the setup is arranged. In Figure 10b, the induction coil was removed, while the IR camera positions including area 1 and 2 have not been moved/modified. After removing the induction coil, the temperature of area 1 is 36.8 °C and that of area 2 is 31.0 °C.

The comparison of the temperatures of the undoped and doped samples from area 1 and area 2 before and after exposure to AMF implies that there is a possibility of the doped layer sample being heated by the hysteresis effect slightly. Heat can also be detected on both samples while the table temperature (19.8 °C) is still close to its value at the start (20.1 °C in Figure 10a) before removing the induction coil. It should be noted that, during exposure to AMF, the temperature (62.8 °C) of area 1 is less than the temperature (86.6 °C) of area 2. Still, after the removal of the induction coil, area 1, where the doped layer is located, is showing a higher temperature (36.8 °C) than the temperature in area 2, corresponding to the undoped layer (31.0 °C). The temperature (36.8 °C) achieved by the doped layer is higher than the LCST (i.e., 32 °C) of the pure poly-NIPA polymer. Currently, the difference in the temperature between the undoped and doped layer is 5.8 °C. A higher temperature change in the doped layer may be achieved by exposing the samples for a longer duration and to the same induction heating conditions for both the undoped and doped samples (as, here, area 1 and area 2 temperatures are different during exposure to AMF). Additionally, it should be kept in consideration that there is a possibility that the difference in the temperature of the undoped and doped layers is not solely due to the exposure to the magnetic field but also due to the different thermal emissivity of the layers.

The main purpose of this study is to achieve the functionalisation of the photopolymer layers and achieve sensitivity to AMF. This characteristic will, at the next stage, be used to produce changes in the properties (diffraction efficiency and/or colour) of the recorded holograms in the presence of AMF. Our work as a proof of concept suggests that the spatial mapping of strong AMF by holographic sensors may be feasible since the observed response was detected with magnetic field strengths approximately ten times smaller than the one typically used in medical MRI systems. Further in-depth studies will be beneficial on the optimisation of material (e.g., suitable-size MNPs and their concentration), recording conditions (e.g., spatial frequency, optimum recording intensity and exposure time), layers thickness for achieving higher diffraction efficiency gratings and, additionally, to optimise the conditions (frequency, field strength, power) of AMF.

## 5. Conclusions

The recording of the volume transmission and reflection holographic gratings is reported here for the first time in photopolymer magnetic nanocomposite (NIPA-photopolymer doped with MNPs). The effect of the nanoparticles’ concentration on the recording of volume holographic gratings was studied. A maximum diffraction efficiency of up to 30% was achieved in pure NIPA. The diffraction efficiency of the doped layers was observed to decrease with the concentration of the nanoparticles; the measured maximum DE was up to 27%, 24% and 22%, respectively, in the case of 0.5, 1 and 2% wt./wt. concentration of MNPs Fe_2_O_3_ Alpha with an average size of 20 to 30 nm. The ability of the magnetic nanocomposites to record high spatial frequency interference patterns was confirmed through the successful recording of reflection holograms.

After holographic recording, the thermal response of the samples was investigated for undoped and doped dry layer samples as well as for additional liquid (solution) samples (composition A, B) (PVA doped with MNPs) through magnetic induction phenomena by exposing them to alternating magnetic field (AMF). A difference of 5.8 °C was reported between the temperature of the undoped and doped dry layers after exposure to AMF, showing a possibility of the doped layer sample being heated by a magnetic field. In the case of the liquid samples (composition A doped with Fe_3_O_4_ 20 nm MNPs and composition B doped with Fe_2_O_3_ Alpha 20–30 nm MNPs), an increase of only approximately 1 to 2 °C was recorded in temperature by exposing them to AMF in a low-power induction system (Easy Heat 2.4 kW, Ambrell; magnetic field strength—0.06 mT; power—54 W)). For the same composition A, a much larger increase was reported in the temperature of 36 °C after a 300 s exposure time by exposure to AMF from the high-power induction system (EASYHEAT 0224, Ambrell; magnetic field strength—111 mT, power 2.12 kW). The high rise in the temperature of the same sample composition A indicates that the heat generation in MNPs has a dependence on the magnetic field, as expected.

The results reported here confirm that holographic recording is possible in the photopolymer magnetic nanocomposite material, and, additionally, such magnetic nanocomposites can be heated locally in the AMF through induction heating.

## Figures and Tables

**Table 1:** Composition of liquid samples (A, B); they were exposed to magnetic induction under alternating magnetic field.

Function/Role	Chemical Component	Liquid (Solution) Sample	
		(A) PVA Doped with Fe_3_O_4_ (20 nm)	(B) PVA Doped with Fe_2_O_3_ (20–30 nm)
Host solution (PVA Binder)	(PVA 10% wt/vol), mL	4	4
Nano dopants (magnetic nanoparticles-MNPs)	Fe_3_O_4_ (20 nm), g	0.1122	-
	Fe_2_O_3_ (20–30 nm), g	-	0.1122

**Table 2:** Composition of dry layer samples (C, D, E). The dry layer samples, (C)-pure NIPA-based photopolymer and (D)-magnetic nanocomposite (NIPA-based photopolymer doped with MNPs), are used for recording volume transmission holographic gratings, while sample (E)-magnetic nanocomposite (NIPA-based photopolymer doped with MNPs) is used for recording Denisyuk reflection holographic gratings.

Function/Role	Chemical Component *	Dry Layer Samples								
		(C)-Trans Pure NIPA	(D)-Trans Nanocomposite (MNPs in NIPA)					(E)-Reflect Nanocomposite (MNPs in NIPA)		
Binder	PVA 10% wt./vol, mL	16	16					16		
Monomer	NIPA, g	0.2	0.2					0.2		
Cross-linker	BA, g	0.15	0.15					0.15		
Free-radical generator	NPG, g	0.04	0.04					0.04		
Sensitising dye	Er B 0.11% wt./vol, mL	2	2					2		
Plasticiser/free radical scavenger	Glycerol, mL	0.2	0.2					1		
Chain transfer agent	CA, g	-	-					0.08		
magnetic nanoparticles (MNP)	Fe_2_O_3_ Alpha 20–30 nm	-	**concentrations of MNPs in dry layer** **(% wt./wt.)**							
			0.5	1	2	5	10	0	0.5	1
	Fe_3_O_4_ 20 nm	-	0.5	1	2	5	10	-		

* Polyvinyl alcohol (PVA), N-isopropyl acrylamide (NIPA), N,N-Methylene bisacrylamide (BA), N-Phenylglycine (NPG), Erythrosine B (Er B), Glycerol, citric acid (CA), Maghemite (Fe_2_O_3_), Magnetite (Fe_3_O_4_).

## Data Availability

Not applicable.

## Supplementary Materials

The following supporting information can be downloaded at: https://www.mdpi.com/article/10.3390/polym14091858/s1, Figure S1: Physical pictures of the photosensitive magnetic nanocomposite layers having MNPs Fe_2_O_3_-Alpha 20–30 nm before UV exposure (a) 1 wt.%, (b) 2 wt.%, (c) 5 wt.%, (d) transmission hologram recorded in the magnetic nanocomposite layer and (e) 1 wt.%, (f) 2 wt.%, (g) 5 wt.% are after UV exposure. Layers were exposed for 100 s in Dymax UV-curing system (model ECE-200) and UV intensity of 60 mW/cm^2^; Figure S2: Physical pictures of the layers used in recording Denisyuk reflection holograms. (a) Pure NIPA photopolymer, magnetic nanocomposites having (b) 0.5 wt.%, (c) 1 wt.% of Fe_2_O_3_ Alpha 20–30 nm; Figure S3: UV–Vis extinction (scattering plus absorption) spectrum of solid layers of photopolymer samples doped with (a) Fe_2_O_3_ and (b) Fe_3_O_4_ nanoparticles; Figure S4: Morphology of the transmission holographic gratings studied by atomic force microscopy (AFM); the gratings were recorded in magnetic nanocomposite layer having MNPs Fe_2_O_3_-Alpha 20–30 nm. The grating period obtained is about 1.25 μm, matching the grating period value for the recording spatial frequency of approximately 800 lines/mm used in transmission holographic recording.

## References

[1] Fernández E., García C., Pascual I., Ortuño M., Gallego S., Beléndez A. (2006). Optimization of a Thick Polyvinyl Alcohol-Acrylamide Photopolymer for Data Storage Using a Combination of Angular and Peristrophic Holographic Multiplexing. Appl. Opt..

[2] Malallah R., Li H., Kelly D.P., Healy J.J., Sheridan J.T. (2017). A Review of Hologram Storage and Self-Written Waveguides Formation in Photopolymer Media. Polymers.

[3] Xiao J., Liu J., Lv Z., Shi X., Han J. (2019). On-axis near-eye display system based on directional scattering holographic waveguide and curved goggle. Opt. Express.

[4] Cem A., Hedili M.K., Ulusoy E., Urey H. (2020). Foveated near-eye display using computational holography. Sci. Rep..

[5] Bigler C.M., Blanche P.-A., Sarma K. (2018). Holographic waveguide heads-up display for longitudinal image magnification and pupil expansion. Appl. Opt..

[6] Hong K., Yeom J., Jang C., Hong J., Lee B. (2014). Full-color lens-array holographic optical element for three-dimensional optical see-through augmented reality. Opt. Lett..

[7] Lee Y., Lee K. (2020). Effective Light Beam Modulation by Chirp IDT on a Suspended LiNbO_3_ Membrane for 3D Holographic Displays. Sensors.

[8] Trapp J.M., Decker M., Petschulat J., Pertsch T., Jabbour T.G. (2018). Design of a 2 diopter holographic progressive lens. Opt. Express.

[9] Lloret T., Navarro-Fuster V., Ramírez M., Ortuño M., Neipp C., Beléndez A., Pascual I. (2018). Holographic Lenses in an Environment-Friendly Photopolymer. Polymers.

[10] Shen Z., Lan T., Wang L., Ni G. (2014). Color demultiplexer using angularly multiplexed volume holograms as a receiver optical end for VLC based on RGB white LED. Opt. Commun..

[11] Toal V., Blanche P.A. (2020). Holographic Security in Optical Holography: Materials, Theory and Applications.

[12] Akbari H., Naydenova I., Ahmed H., McCormack S., Martin S. (2017). Development and testing of low spatial frequency holographic concentrator elements for collection of solar energy. Sol. Energy.

[13] Marín-Sáez J., Atencia J., Chemisana D., Collados M.-V. (2016). Characterization of volume holographic optical elements recorded in Bayfol HX photopolymer for solar photovoltaic applications. Opt. Express.

[14] Ferrara M.A., Striano V., Coppola G. (2019). Volume Holographic Optical Elements as Solar Concentrators: An Overview. Appl. Sci..

[15] Vorndran S.D., Chrysler B., Wheelwright B., Angel R., Holman Z., Kostuk R. (2016). Off-axis holographic lens spectrum-splitting photovoltaic system for direct and diffuse solar energy conversion. Appl. Opt..

[16] Květoň M., Lédl V., Havránek A., Fiala P. (2010). Photopolymer for Optical Holography and Holographic Interferometry. Macromol. Symp..

[17] Georges M., Blanche P.A. (2020). Holographic Interferometry: From History to Modern Applications.

[18] Nazarova D., Mateev G., Nedelchev L., Stoykova E., Blagoeva B., Berberova N., Hong K., Park J. (2021). Polarization holographic gratings with enhanced parameters recorded in azopolymer based nanocomposite materials. Optik.

[19] Nedelchev L., Ivanov D., Berberova N., Strijkova V., Nazarova D. (2018). Polarization holographic gratings with high diffraction efficiency recorded in azopolymer PAZO. Opt. Quantum Electron..

[20] Chen W., Zhao Z., Wang C., Li H., Wei R., Zhang S., Peng Z., Liu Y., Wang Q., Mu Q. (2019). Linear polarization grating combining a circular polarization grating with a special cycloidal diffractive quarter waveplate. Opt. Express.

[21] Zhou K., Geng Y., Liu H., Wang S., Mao D., Yu D. (2017). Improvement of holographic sensing response in substrate-free acrylamide photopolymer. Appl. Opt..

[22] Fuchs Y., Soppera O., Mayes A.G., Haupt K. (2013). Holographic Molecularly Imprinted Polymers for Label-Free Chemical Sensing. Adv. Mater..

[23] Hsiao V.K.S., Kirkey W.D., Chen F., Cartwright A.N., Prasad P.N., Bunning T.J. (2005). Organic Solvent Vapor Detection Using Holographic Photopolymer Reflection Gratings. Adv. Mater..

[24] Yetisen A.K., Butt H., da Cruz Vasconcellos F., Montelongo Y., Davidson C.A.B., Blyth J., Chan L., Carmody J.B., Vignolini S., Steiner U. (2014). Light-Directed Writing of Chemically Tunable Narrow-Band Holographic Sensors. Adv. Opt. Mater..

[25] Yetisen A.K., Naydenova I., da Cruz Vasconcellos F., Blyth J., Lowe C.R. (2014). Holographic Sensors: Three-Dimensional Analyte-Sensitive Nanostructures and Their Applications. Chem. Rev..

[26] Grogan C., McGovern F.R., Staines R., Amarandei G., Naydenova I. (2021). Cantilever-Based Sensor Utilizing a Diffractive Optical Element with High Sensitivity to Relative Humidity. Sensors.

[27] Naydenova I., Blanche P.A. (2020). Holographic Sensors in Optical Holography: Materials, Theory and Applications.

[28] Jurbergs D., Bruder F.-K., Deuber F., Fäcke T., Hagen R., Hönel D., Rölle T., Weiser M.-S., Volkov A. (2009). New recording materials for the holographic industry. Proceedings of the SPIE 7233, Practical Holography XXIII: Materials and Applications.

[29] Cody D., Gul S., Mikulchyk T., Irfan M., Kharchenko A., Goldyn K., Martin S., Mintova S., Cassidy J., Naydenova I. (2018). Self-processing photopolymer materials for versatile design and fabrication of holographic sensors and interactive holograms. Appl. Opt..

[30] Kabilan S., Blyth J., Lee M.C., Marshall A.J., Hussain A., Yang X.-P., Lowe C.R. (2004). Glucose-sensitive holographic sensors. J. Mol. Recognit..

[31] Yetisen A.K., Montelongo Y., da Cruz Vasconcellos F., Martinez-Hurtado J.L., Neupane S., Butt H., Qasim M.M., Blyth J., Burling K., Carmody J.B. (2014). Reusable, Robust, and Accurate Laser-Generated Photonic Nanosensor. Nano Lett..

[32] Marshall A.J., Blyth J., Davidson C.A.B., Lowe C.R. (2003). pH-Sensitive Holographic Sensors. Anal. Chem..

[33] Liu H., Yu D., Zhou K., Wang S., Luo S., Li L., Wang W., Song Q. (2018). Novel pH-sensitive photopolymer hydrogel and its holographic sensing response for solution characterization. Opt. Laser Technol..

[34] Rai K., Fontecchio A.K. (2006). Optimization of Pressure Response in HPDLC Gratings Based on Polymer Composition. Mol. Cryst. Liq. Cryst..

[35] Ermold M.L., Rai K., Fontecchio A.K. (2005). Hydrostatic pressure response of polymer-dispersed liquid crystal gratings. J. Appl. Phys..

[36] Yu D., Liu H., Mao D., Geng Y., Wang W., Sun L., Lv J. (2015). Holographic humidity response of slanted gratings in moisture-absorbing acrylamide photopolymer. Appl. Opt..

[37] Mikulchyk T., Martin S., Naydenova I. (2014). Investigation of the sensitivity to humidity of an acrylamide-based photopolymer containing N-phenylglycine as a photoinitiator. Opt. Mater..

[38] Mikulchyk T., Walshe J., Cody D., Martin S., Naydenova I. (2017). Humidity and temperature induced changes in the diffraction efficiency and the Bragg angle of slanted photopolymer-based holographic gratings. Sens. Actuators B Chem..

[39] Liu H., Yu D., Zhou K., Mao D., Liu L., Wang H., Wang W., Song Q. (2016). Temperature-induced spectrum response of volume grating as an effective strategy for holographic sensing in acrylamide polymer part I: Sensing. Appl. Opt..

[40] Yetisen A.K., Montelongo Y., Qasim M.M., Butt H., Wilkinson T.D., Monteiro M.J., Yun S.H. (2015). Photonic Nanosensor for Colorimetric Detection of Metal Ions. Anal. Chem..

[41] Madrigal González B., Christie G., Davidson C.A.B., Blyth J., Lowe C.R. (2005). Divalent metal ion-sensitive holographic sensors. Anal. Chim. Acta.

[42] Bianco G., Ferrara M.A., Borbone F., Zuppardi F., Roviello A., Striano V., Coppola G. (2015). Volume holographic gratings as optical sensor for heavy metal in bathing waters. Proceedings of the SPIE 9506, Optical Sensors 95062B.

[43] Cody D., Gribbin S., Mihaylova E., Naydenova I. (2016). Low-Toxicity Photopolymer for Reflection Holography. ACS Appl. Mater. Interfaces.

[44] Mikulchyk T., Martin S., Naydenova I. (2017). N-isopropylacrylamide-based photopolymer for holographic recording of thermosensitive transmission and reflection gratings. Appl. Opt..

[45] Yu D., Liu H., Mao D., Geng Y., Wang W., Sun L., Lv J. (2015). Enhancement of spectrum strength in holographic sensing in nanozeolites dispersed acrylamide photopolymer. Opt. Express.

[46] Naydenova I., Grand J., Mikulchyk T., Martin S., Toal V., Georgieva V., Thomas S., Mintova S. (2015). Hybrid Sensors Fabricated by Inkjet Printing and Holographic Patterning. Chem. Mater..

[47] Leite E. (2010). Photopolymerizable Nanocomposites for Holographic Applications. Ph.D. Thesis.

[48] Naydenova I., Toal V., Valtchev V., Mintova S., Tsapatsis M. (2009). Nanoparticle Doped Photopolymers for Holographic Applications in Ordered Porous Solids Recent Advances and Prospects.

[49] Tomita Y., Urano H., Fukamizu T., Kametani Y., Nishimura N., Odoi K. (2016). Nanoparticle-polymer composite volume holographic gratings dispersed with ultrahigh-refractive-index hyperbranched polymer as organic nanoparticles. Opt. Lett..

[50] Tomita Y., Furushima K., Ochi K., Ishizu K., Tanaka A., Ozawa M., Hidaka M., Chikama K. (2006). Organic nanoparticle (hyperbranched polymer)-dispersed photopolymers for volume holographic storage. Appl. Phys. Lett..

[51] Suzuki N., Tomita Y., Kojima T. (2002). Holographic recording in TiO2 nanoparticle-dispersed methacrylate photopolymer films. Appl. Phys. Lett..

[52] Sánchez C., Escuti M.J., van Heesch C., Bastiaansen C.W.M., Broer D.J., Loos J., Nussbaumer R. (2005). TiO2 Nanoparticle-Photopolymer Composites for Volume Holographic Recording. Adv. Funct. Mater..

[53] Sakhno O.V., Goldenberg L.M., Stumpe J., Smirnova T.N. (2007). Surface modified ZrO_2_ and TiO_2_ nanoparticles embedded in organic photopolymers for highly effective and UV-stable volume holograms. Nanotechnology.

[54] Suzuki N., Tomita Y., Ohmori K., Hidaka M., Chikama K. (2006). Highly transparent ZrO_2_ nanoparticle-dispersed acrylate photopolymers for volume holographic recording. Opt. Express.

[55] Tomita Y., Nishibiraki H. (2003). Improvement of holographic recording sensitivities in the green in SiO2 nanoparticle-dispersed methacrylate photopolymers doped with pyrromethene dyes. Appl. Phys. Lett..

[56] Suzuki N., Tomita Y. (2003). Diffraction Properties of Volume Holograms Recorded in SiO_2_ Nanoparticle-Dispersed Methacrylate Photopolymer Films. Jpn. J. Appl. Phys..

[57] Kim W.S., Jeong Y.-C., Park J.-K. (2006). Nanoparticle-induced refractive index modulation of organic-inorganic hybrid photopolymer. Opt. Express.

[58] Xue X., Hai F., Gao L., He F., Li C., Li Y., Huang M. (2013). Effect of nanoparticle diameter on the holographic properties of gold nanoparticle dispersed acrylate photopolymer films. Optik.

[59] Naydenova I., Sherif H., Mintova S., Martin S., Toal V. Holographic recording in nanoparticle-doped photopolymer. Proceedings of the SPIE 6252, Holography 2005: International Conference on Holography, Optical Recording, and Processing of Information.

[60] Cody D., Mihaylova E., O’Neill L., Babeva T., Awala H., Retoux R., Mintova S., Naydenova I. (2014). Effect of zeolite nanoparticles on the optical properties of diacetone acrylamide-based photopolymer. Opt. Mater..

[61] Naydenova I., Leite E., Babeva T., Pandey N., Baron T., Yovcheva T., Sainov S., Martin S., Mintova S., Toal V. (2011). Optical properties of photopolymerizable nanocomposites containing nanosized molecular sieves. J. Opt..

[62] Leite E., Naydenova I., Mintova S., Leclercq L., Toal V. (2010). Photopolymerizable nanocomposites for holographic recording and sensor application. Appl. Opt..

[63] Leite E., Babeva T.Z., Ng E.-P., Toal V., Mintova S., Naydenova I. (2010). Optical Properties of Photopolymer Layers Doped with Aluminophosphate Nanocrystals. J. Phys. Chem. C.

[64] Pierini F., Guglielmelli A., Urbanek O., Nakielski P., Pezzi L., Buda R., Lanzi M., Kowalewski T.A., De Sio L. (2020). Thermoplasmonic-Activated Hydrogel Based Dynamic Light Attenuator. Adv. Opt. Mater..

[65] Espinosa A., Kolosnjaj-Tabi J., Abou-Hassan A., Plan Sangnier A., Curcio A., Silva A.K.A., Di Corato R., Neveu S., Pellegrino T., Liz-Marzán L.M. (2018). Magnetic (Hyper)Thermia or Photothermia? Progressive Comparison of Iron Oxide and Gold Nanoparticles Heating in Water, in Cells, and In Vivo. Adv. Funct. Mater..

[66] Anik M.I., Hossain M.K., Hossain I., Mahfuz A.M.U.B., Rahman M.T., Ahmed I. (2021). Recent progress of magnetic nanoparticles in biomedical applications: A review. Nano Sel..

[67] Satarkar N.S., Biswal D., Hilt J.Z. (2010). Hydrogel nanocomposites: A review of applications as remote-controlled biomaterials. Soft Matter.

[68] Satarkar N.S., Zhang W., Eitel R.E., Hilt J.Z. (2009). Magnetic hydrogel nanocomposites as remote-controlled microfluidic valves. Lab Chip.

[69] Liu H., Yu D., Zhou K., Wang S., Luo S., Wang W., Song Q. (2017). Improvement of temperature-induced spectrum characterization in a holographic sensor based on N-isopropylacrylamide photopolymer hydrogel. Appl. Opt..

[70] Irfan M., Mikulchyk T., Martin S., Naydenova I. (2018). Investigation of temperature response of photopolymer material used for holographic sensor. Proceedings of the 2018 15th International Bhurban Conference on Applied Sciences and Technology (IBCAST).

[71] Irfan M., Martin S., Naydenova I. (2021). Temperature-Sensitive Holograms with Switchable Memory. Adv. Photo Res..

[72] Kumar C.S.S.R., Mohammad F. (2011). Magnetic nanomaterials for hyperthermia-based therapy and controlled drug delivery. Adv. Drug Deliv. Rev..

[73] Irfan M., Martin S., Naydenova I. Study of effect of magnetic nanoparticles properties on hologram recording capability in photopolymer nanocomposite for development of holographic sensor/actuator. Proceedings of the SPIE Volume 11081, SPIE Nanoscience + Engineering.

[74] Kogelnik H. (1969). Coupled Wave Theory for Thick Hologram Gratings. Bell Syst. Tech. J..

[75] Hervault A., Thanh N.T.K. (2014). Magnetic nanoparticle-based therapeutic agents for thermo-chemotherapy treatment of cancer. Nanoscale.

[76] Abenojar E.C., Wickramasinghe S., Bas-Concepcion J., Samia A.C.S. (2016). Structural effects on the magnetic hyperthermia properties of iron oxide nanoparticles. Progress in Natural Science. Mater. Int..

[77] Akbarzadeh A., Samiei M., Davaran S. (2012). Magnetic nanoparticles: Preparation, physical properties, and applications in biomedicine. Nanoscale Res. Lett..

[78] Tong S., Quinto C.A., Zhang L., Mohindra P., Bao G. (2017). Size-Dependent Heating of Magnetic Iron Oxide Nanoparticles. ACS Nano.

[79] Soo Choi H., Liu W., Misra P., Tanaka E., Zimmer J.P., Itty Ipe B., Bawendi M.G., Frangioni J.V. (2007). Renal clearance of quantum dots. Nat. Biotechnol..

[80] Murray C.B., Norris D.J., Bawendi M.G. (1993). Synthesis and characterization of nearly monodisperse CdE (E = sulfur, selenium, tellurium) semiconductor nanocrystallites. J. Am. Chem. Soc..

[81] Peng X., Manna L., Yang W., Wickham J., Scher E., Kadavanich A., Alivisatos A.P. (2000). Shape control of CdSe nanocrystals. Nature.

[82] Deshmukh M.V., Vaidya A.A., Kulkarni M.G., Rajamohanan P.R., Ganapathy S. (2000). LCST in poly(N-isopropylacrylamide) copolymers: High resolution proton NMR investigations. Polymer.

[83] Frimpong R.A., Fraser S., Zach Hilt J. (2007). Synthesis and temperature response analysis of magnetic-hydrogel nanocomposites. J. Biomed. Mater. Res..

[84] Harabech M., Kiselovs N.R., Maenhoudt W., Crevecoeur G., Roost D.V., Dupré L. (2017). Experimental ex-vivo validation of PMMA-based bone cements loaded with magnetic nanoparticles enabling hyperthermia of metastatic bone tumors. AIP Adv..

