# Supplementary material for: A Magnetic Nanoparticle-Doped Photopolymer for Holographic Recording
Source: Polymers (Basel). 2022 Apr 30;14(9):1858. doi: 10.3390/polym14091858 (PMC9102196; doi:10.3390/polym14091858)
Supplement: Supplementary file 1 [file polymers-14-01858-s001.zip › polymers-1712357-supplementary.pdf]

polymers

MDPI

Supplementary Materials

A Magnetic Nanoparticle-Doped Photopolymer for Holographic Recording

Muhammad Irfan, Suzanne Martin, Muhannad Ahmed Obeidi, Scott Miller [3], Frank Kuster, Dermot Brabazon and Izabela Naydenova

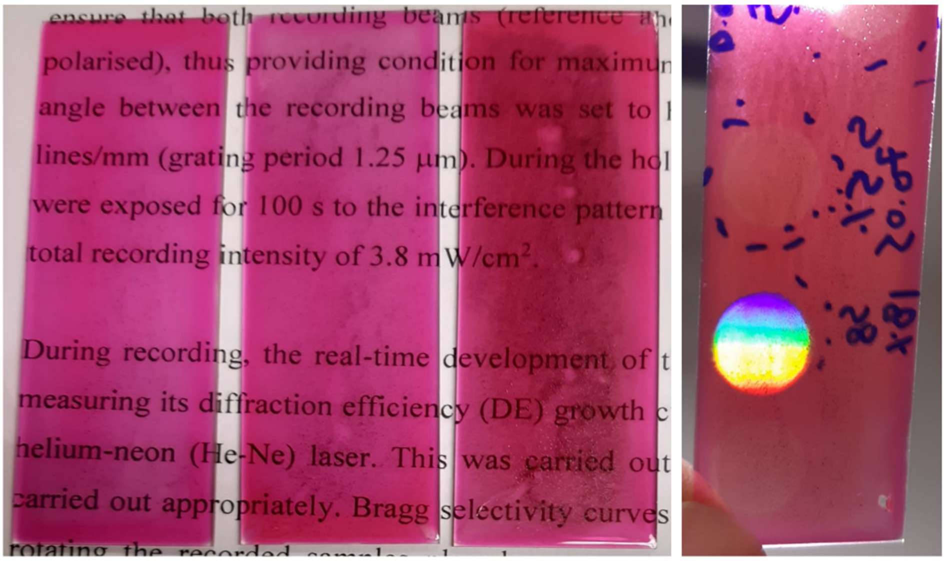

(a) B.UV Fe_2O_3 20-30 nm 1 wt.% (b) B.UV Fe_2O_3 20-30 nm 2 wt.% (c) B.UV Fe_2O_3 20-30 nm 5 wt.% (d) Transmission hologram recorded in nanocomposite layer

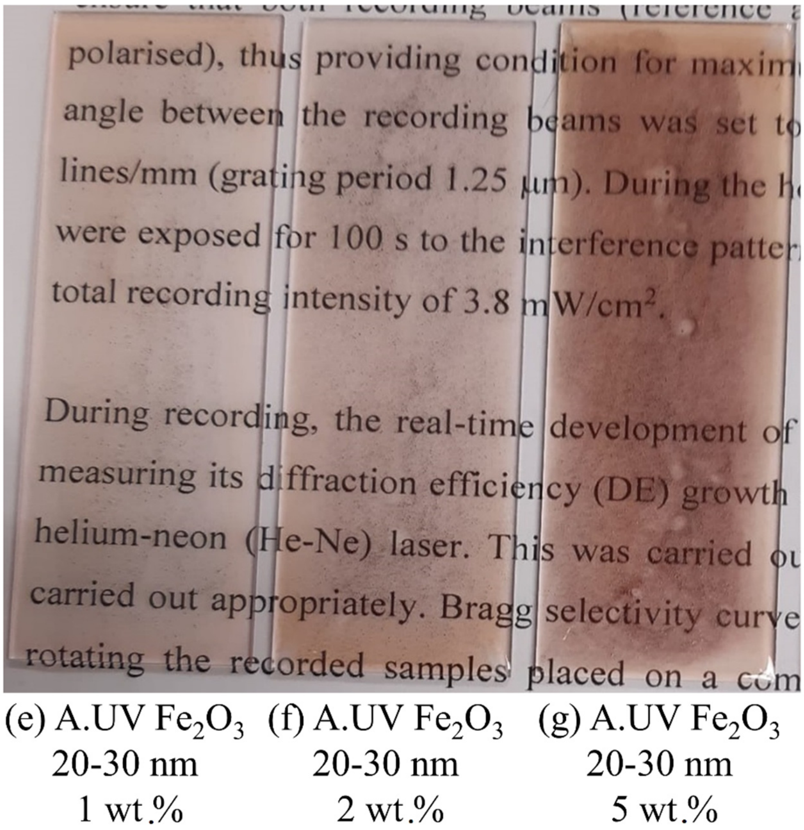

Figure S1. Physical pictures of the photosensitive magnetic nanocomposite layers having MNPs Fe_2O_3-Alpha 20–30 nm before UV exposure (**a**) 1 wt.%, (**b**) 2 wt.%, (**c**) 5 wt.%, (**d**) transmission hologram recorded in the magnetic nanocomposite layer and (**e**) 1 wt.%, (**f**) 2 wt.%, (**g**) 5 wt.% are after UV exposure. Layers were exposed for 100 s in Dymax UV-curing system (model ECE-200) and UV intensity of 60 mW/cm^2.

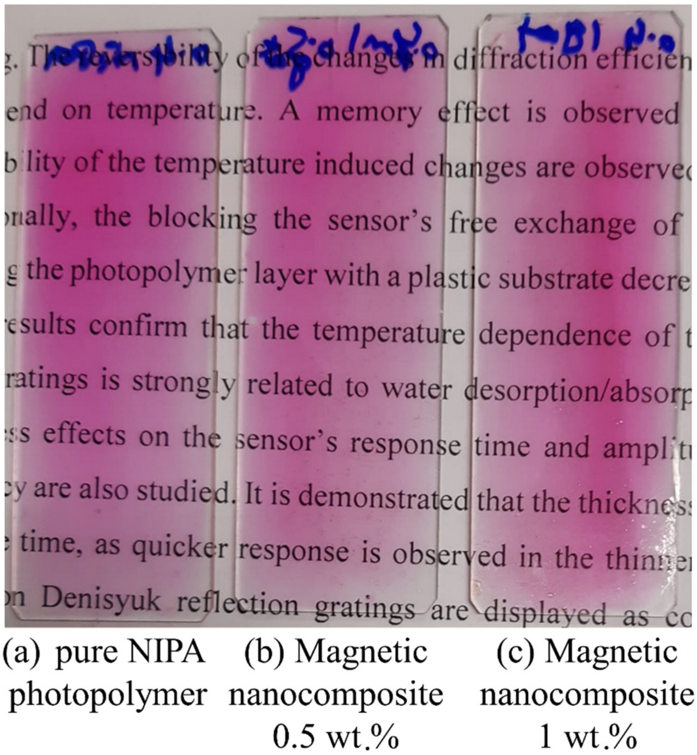

Figure S2. Physical pictures of the layers used in recording Denisyuk reflection holograms. (a) Pure NIPA photopolymer, magnetic nanocomposites having (b) 0.5 wt.%, (c) 1 wt.% of Fe_2O_3 Alpha 20–30 nm.

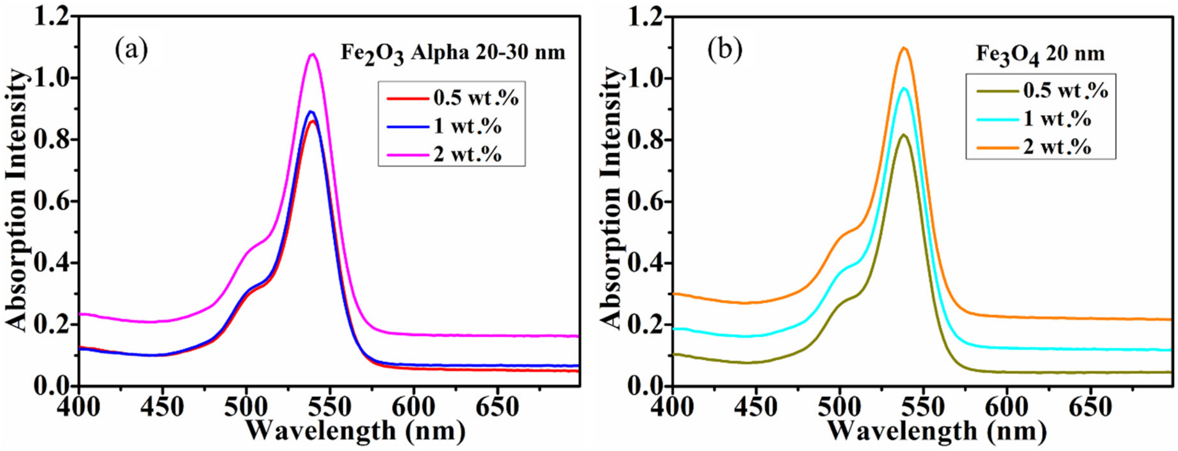

Figure S3. UV–Vis extinction (scattering plus absorption) spectrum of solid layers of photopolymer samples doped with a) Fe_2O_3 and b) Fe_3O_4 nanoparticles.

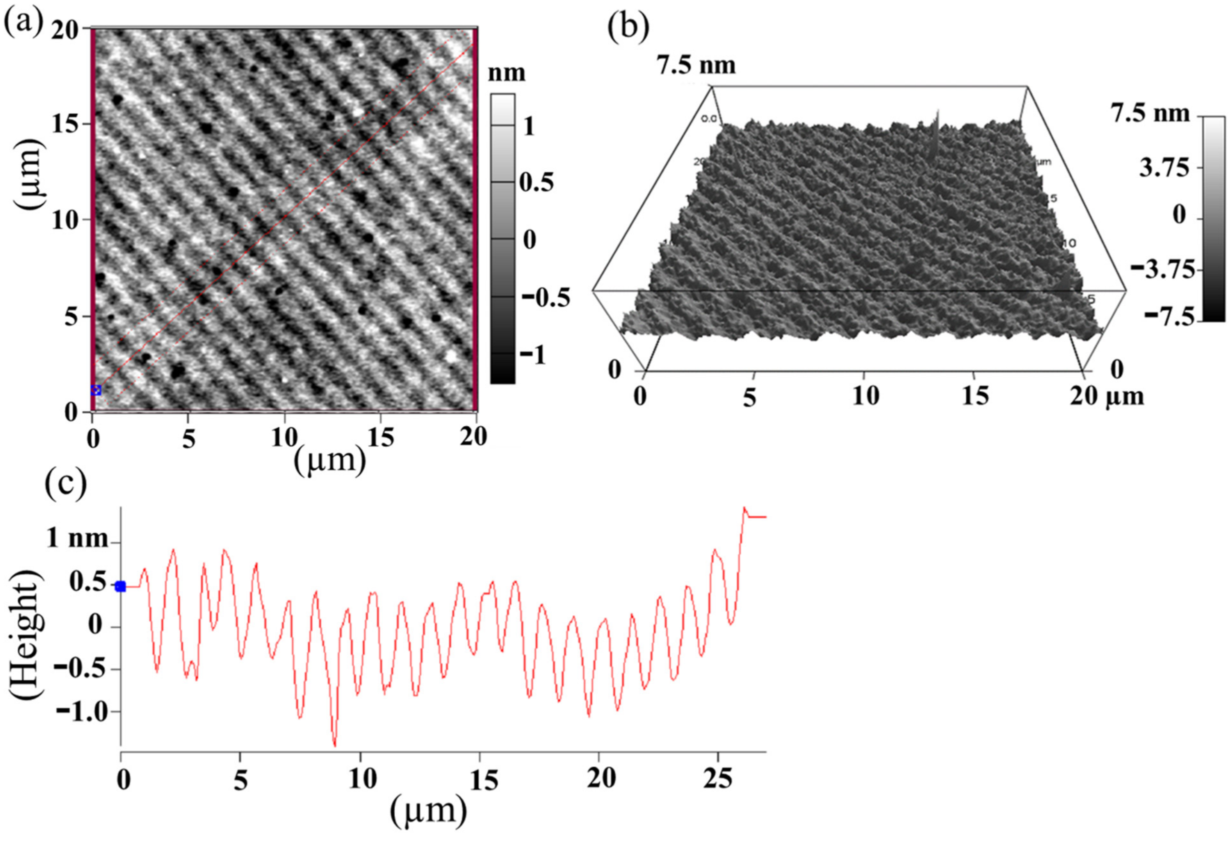

Figure S4. Morphology of the transmission holographic gratings studied by atomic force microscopy (AFM); the gratings were recorded in magnetic nanocomposite layer having MNPs Fe_2O_3-Alpha 20–30 nm. The grating period obtained is about 1.25 μm, matching the grating period value for the recording spatial frequency of approximately 800 lines/mm used in transmission holographic recording.
